# Identification of DNA Binding Motifs of the *Mycobacterium tuberculosis* PhoP/PhoR Two-Component Signal Transduction System

**DOI:** 10.1371/journal.pone.0042876

**Published:** 2012-08-07

**Authors:** Mena Cimino, Christophe Thomas, Amine Namouchi, Sarah Dubrac, Brigitte Gicquel, Deshmukh N. Gopaul

**Affiliations:** 1 Unité de Génétique Mycobacterienne, Institut Pasteur, Paris, France; 2 Unité de Plasticité du Génome bactérien, Institut Pasteur, CNRS UMR 3525, Paris, France; 3 Unité de Recherche Biologie des Bactéries pathogènes à Gram-positif, Institut Pasteur, Paris, France; Johns Hopkins University School of Medicine, United States of America

## Abstract

**Background:**

The *Mycobacterium tuberculosis* PhoP/PhoR two-component signal transduction system controls the expression of about 2% of the genome and plays a major role in pathogenicity. However, its regulon has not been well characterized.

**Methodology/Principal Findings:**

The binding site of PhoP transcription regulator was identified in the upstream regions of *msl3, pks2, lipF* and *fadD21* genes, by using gene fusions, electrophoretic mobility shift assays and DNase I footprinting experiments. A consensus sequence for PhoP binding was deduced. It consists of two direct repeats, DR1/DR2, associated with a third repeat, DR3, important in some cases for PhoP binding to DR1/DR2 but located at a variable distance from these direct repeats. DR1/DR2 and DR3 consensus sequences were used to screen the whole-genome sequence for other putative binding sites potentially corresponding to genes directly regulated by PhoP. The identified 87 genes, encoding transcription regulators, and proteins involved in secondary metabolites biosynthesis, transport and catabolism are proposed to belong to the PhoP regulon.

**Conclusions/Significance:**

A consensus sequence derived from the analysis of PhoP binding to four gene promoter regions is proposed. We show for the first time the involvement of a third direct repeat motif in this binding reaction. The consensus sequence was instrumented to study the global regulation mediated by PhoP in *M. tuberculosis*. This analysis leads to the identification of several genes that are potentially regulated by this key player.

## Introduction

*Mycobacterium tuberculosis* (MTB), the causal agent of tuberculosis in humans, is one of the leading causes of mortality due to a single infectious agent. MTB is a successful intracellular pathogen that can adapt to changing environmental conditions within the host. It can infect different cell types, in which it may replicate or remain dormant for years. Differential expression of MTB genes is observed during the infection of various cells of the immune system, such as dendritic cells, macrophages and alveolar epithelial cells, during disease progression [1], [2], [3], [4]. We have previously shown that bacterial stress responses are more strongly induced in dendritic cells, whereas genes encoding ribosomal proteins are overexpressed, indicating bacterial multiplication, in macrophages [5].

As in other prokaryotes, two-component signal transduction systems (TCS) are key elements of the adaptive response to various stimuli in the tubercle bacillus. TCS contain an environmentally sensitive histidine kinase (HK) and a response regulator (RR) that is activated by the cognate HK. These systems play a major role in bacterial responses to changing growth contexts. The MTB genome encodes 11 TCS [6]. This relatively small number of TCS probably reflects the intracellular lifestyle of MTB, as the cell environment is less variable than that confronted by soil bacteria, or a certain degree of overlap in signal processing.

The MTB *phoP* gene has been shown to encode one of the components of a TCS (PhoP/R) playing a major role in virulence [7]. Inactivation of *phoP* results in an attenuated mutant unable to replicate in animal models and in cells cultured *in vitro*, but able to persist, unlike auxotrophs. This led to the construction of live candidate vaccines against tuberculosis based on the inactivation of *phoP* [8]. Analyses of the genomes of known avirulent strains, such as the BCG vaccine or the H37Ra attenuated clinical variant, showed mutations in *phoP* or *phoR* resulting in a loss of function, thus confirming the role of *phoP*/*phoR* in virulence [9], [10], [11].

The MTB PhoP regulator belongs to the PhoB/OmpR subfamily, the largest one among response regulators. Members of this subfamily have two domains, an N-terminal regulatory domain and a C-terminal DNA-binding domain (also called the effector domain). Many members of this subfamily have been studied in detail, and most reports have indicated that these response regulators bind DNA as a dimer recognizing tandem repeat binding sites at the -35 region of the regulated promoter [12]. The *Bacillus subtilis* TCS PhoP/PhoR, which belongs to this subfamily, senses phosphate. It is activated in conditions in which phosphate is limited. It regulates the expression of 31 genes involved in phosphate utilization [13]. However, in *Salmonella enterica typhimurium*, PhoP/PhoQ responds to other stimuli, such as Mg^2+^ concentration [14], low pH [15], [16] and antibacterial peptides [17], [18], thereby regulating genes involved in Mg^2+^ homeostasis and virulence [19]. The stimuli sensed by the MTB PhoP/PhoR TCS are unknown.

Efforts have been made to identify genes regulated by this PhoP/PhoR TCS, by comparing the transcriptomes of *phoP* null mutants and wild-type parental strains. Two studies identified a large number of genes positively or negatively regulated by PhoP, 70 of which were upregulated [20], [21]. However, these two studies identified different, though overlapping, sets of regulated genes. Differences could also be due to the use of two different strains in the two studies (H37Rv for one study and MT103 for the other one), or to the lack of knowledge of the metabolites acting as stimuli for this TCS, resulting in non optimal experimental conditions.

Many of the genes upregulated by PhoP are involved in general or lipid metabolism, substrate transport across the plasma membrane and the synthesis of regulators, such as DosR, controlling dormancy. *phoP* null mutants display deficiencies in the synthesis of sulfatides and diacyl and polyacyl trehaloses. *msl3*, a polyketide beta-ketoacyl synthase gene, is involved in the synthesis of polyacyl trehaloses, and the *pks2* and *mmpl8* genes are involved in sulfatide synthesis [21], [22]. The disruption of *pks2* generated a sulfolipid-deficient mutant that was unable to synthesize hydroxyphthioceranic and phthioceranic acids [23]. The *msl3*-disrupted mutant was unable to produce the mycolipanoic and mycolipenic acids required for the synthesis of a major class of polyacylated trehaloses. In the absence of these classes of polyacylated trehaloses, which anchor trehaloses to the cell surface, the mutants grew in bead-like aggregates, with no discernable decrease in either growth rate or in virulence [24]. *phoP* null mutants and H37Ra lack these complex lipids. PhoP/PhoR TCS also controls secretion of the early-secreted 6 kDa antigen (ESAT-6), an important virulence factor and antigenic component of *M. tuberculosis* [9].

The two transcriptomic analyses suggested that the *lipF* and *fadD21* genes were positively regulated by PhoP [20], [21]. The *lipF* gene of MTB encodes an esterase that has been shown to be important in pathogenesis. The insertion of a transposon between the *lipF* promoter region and the transcription start site significantly decreased the ability of the bacterium to grow in mouse lungs [25].

PhoP is autoregulated, as it binds to two adjacent 9-bp direct repeat motifs binding to PhoP in a sequence-specific manner. These motifs are located downstream from the transcription +1 site, contrasting with the models proposed for other members of the PhoB/OmpR TCS family [26]. We investigated PhoP binding sites and their molecular regulation in more detail, by looking for such sites in other promoters thought to be regulated by PhoP on the basis of previous transcriptome analyses.

We first used gene fusions to identify the *msl3*, *pks2*, *lipF* and *fadD21* gene promoters. DNA-affinity electrophoretic mobility shift studies and DNase I protection assays have been used to identify PhoP binding sites. These sites were found to contain tandem repeat sequences displaying similarities that could be used to define a consensus-binding motif. We then carried out whole-genome analysis with these consensus sequences, to identify genes regulated directly by PhoP.

## Results

### Identification of promoter regions

We used *lacZ* as a reporter gene, to measure the activity of *M. tuberculosis* promoters thought to be regulated by PhoP in *M. smegmatis*, a non-pathogenic and fast-growing mycobacterial species.

Several fragments corresponding to upstream and structural gene regions of *pks2* (−86,+60), *msl3* (−282,+28) and *fadD21* (−198, +10) were generated (Table 1). These fragments were inserted upstream from the *lacZ* reporter gene in the pJEM15 *E. coli*-mycobacterial reporter shuttle plasmid. For *lipF*, a 611 bp region (−596, +14) was used, because this region has previously been reported to be required for the upregulation of transcriptional activity in response to exposure to an acid stress. This upregulation has been demonstrated in both pathogenic *M. tuberculosis* and non-pathogenic *M. smegmatis* strains [27]. Cultures of *M. smegmatis* transformed with the empty pJEM15 vector, a positive control, the pJEM31 (PAN) vector containing a promoter sequence, PAN, isolated from *M. paratuberculosis* [28] or recombinant pJEM15 plasmids carrying *lipF*, *pks2*, *msl3* or *fadD21* gene fragments were set up. Aliquots of cultures were collected during the exponential growth phase and beta-galactosidase activities were assessed to determine transcription levels.

**Table 1:** List of primers used in this study.

Fragments	Primers	Sequence (5′-3′ position relative to the translation start site	Fragments size (bp)
**lipF** a	lipFF	(−596) GGCGAATTCGCACTTACCA (−578)	611
	lipFR	(+14) CCAGGCGCACGCACCAAC (−3)	
**lipFa** b	lipFaF	(−596) GGCGAATTCGCACTTACCA (−578)	222
	lipFaR	(−375) ATCAATCGCAAATCTCGCAC (−394)	
**lipFb** b	lipFbF	(−394) GTGCGAGATTTGCGATTGAT (−375)	227
	lipFbR	(−168) CAACCGACCTGCAACACTA (−186)	
**lipFc** b	lipFcF	(−167) GCGGCACTGGCATCGCGCAT (−148)	182
	lipFcR	(+14) CCAGGCGCACGCACCAAC (−3)	
**lipFa2** b	lipFa2F	(−465) CACCGAATGATTGATGG (−449)	89
	lipFa2R	(−377) CAATCGCAAATCTCGCA (−393)	
**lipF** c	lipFDF	(−639) GCCGATATCGGCTTGCGATTC (−619)	189
	lipFDR	(−470) CATCAATCATTCGGTGGCGCG (−450)	
**pks2** a b	pks2F	(−86) TAGCACAGCCGCTTAGAAC (−68)	147
	pks2R	(+60) TCAGCCAACGTCCATGC (+44)	
**pks2c** b	pks2cF	(+10) GTCGGCGGCGTCAGGCACT (+28)	77
	pks2cR	(+86) TAACAGCAACCGGAGTCA (+69)	
**pks2d** b	pks2dF	(−86) TAGCACAGCCGCTTAGAAC (−68)	102
	pks2dR	(+15) GCCGACCCCAAGCCCAAT (−2)	
**pks2** a b	pks2DF	(−137) ACCGCGGGCTGCCCATGCCA (−118)	198
	pks2DR	(+60) TCAGCCAACGTCCATGCA (+43)	
**msl3** a	msl3F	(−282) GTCGAGCAGTGTTGCTACC (−264)	311
	msl3R	(+28) CGACCGATGTGGCGGTTGCGG (+8)	
**msl3a** b	msl3aF	(−282) GTCGAGCAGTGTTGCTACC (−264)	234
	msl3aR	(−49) TACCAACACATTCGGGCTC (−67)	
**msl3b** b	msl3bF	(−67) GAGCCCGAATGTGTTGGTA (−49)	98
	msl3bR	(+30) CGACCGATGTGGCGGTTGCGG (+10)	
**msl3a2** b	msl3a2F	(−269) GCTACCTTAACTTTCCCAG (−251)	82
	msl3a2R	(−188) CGAAGAACACATTATCCAG (−206)	
**msl3** c	msl3DF	(−311) TCACACGCGCGGTGCATGC (−293)	222
	msl3DR	(−90) TCATGGTAGTGGCTCAAGA (−108)	
**fadD21** a b	fadD21F	(−198) GGCCATGGAACGCACGGCTTA (−178)	209
	fadD21R	(+10) GAGTCGGACATTGGTGCTA (−8)	
**fadD21b** b	fadD21bF	(−145) CTGCGTACACCGACTTCGA (−127)	85
	fadD21bR	(−61) GCGATAGCGATGCTGATC (−78)	
**fadD21** c	fadD21DF	(−194) ATGGAACGCACGGCTTAGCAACT (−172)	201
	fadD21DR	(−16) CGGACATTGGTGCTACATTACCG (+7)	

a Fragments used for transcriptional fusions.

b Fragments used for electrophoretic mobility shift assays.

c Fragments used for DNase protection assay.

**Figure 1:**
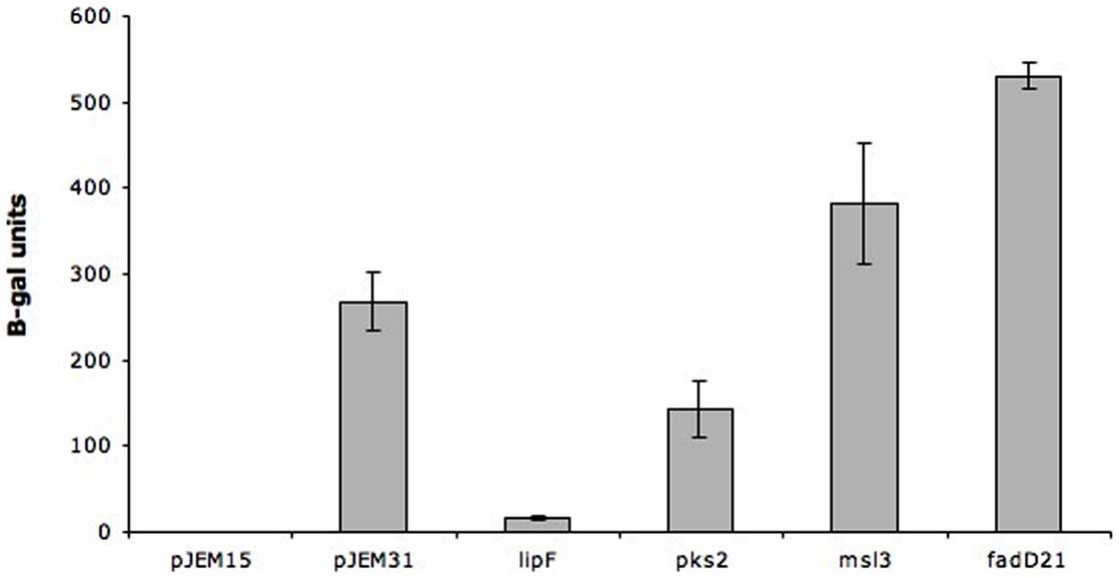
Beta-galactosidase activity in *M. smegmatis* expressing *lacZ* under the control of the promoter regions of the *M. tuberculosis* H37Rv *lipF*, *pks2*, *msl3* and *fadD21* genes. The *lipF*, *pks2*, *msl3* and *fadD21* promoter-*lacZ* fusions carried by the pJEM15 plasmid were introduced into *M. smegmatis*. The positive control is *M. smegmatis* transformed with pJEM31(PAN) containing *lacZ* under the control of a mobile genetic element normalized against *M. smegmatis* transformed with pJEM15 empty vector. The results shown are the mean of two independent experiments.

As indicated in Fig. 1, expression levels were higher for *M. smegmatis* transformed with the recombinant plasmids carrying *pks2*, *msl3, lipF* or *fadD21* gene fusions than for *M. smegmatis* transformed with the empty pJEM15 vector. No significant expression was detected for *M. smegmatis* transformed with the empty pJEM15 vector. With the plasmid pJEM31 (PAN) used as a positive control, we observed expression of similar magnitude as observed for *pks2, msl3* and *fadD21*. A much lower expression was observed for *M. smegmatis* carrying the *lipF* fusion, under the conditions tested. These results demonstrate that there was a promoter present in the various cloned regions in fusion with *lacZ* gene.

### Identification of PhoP binding sites

**Figure 2:**
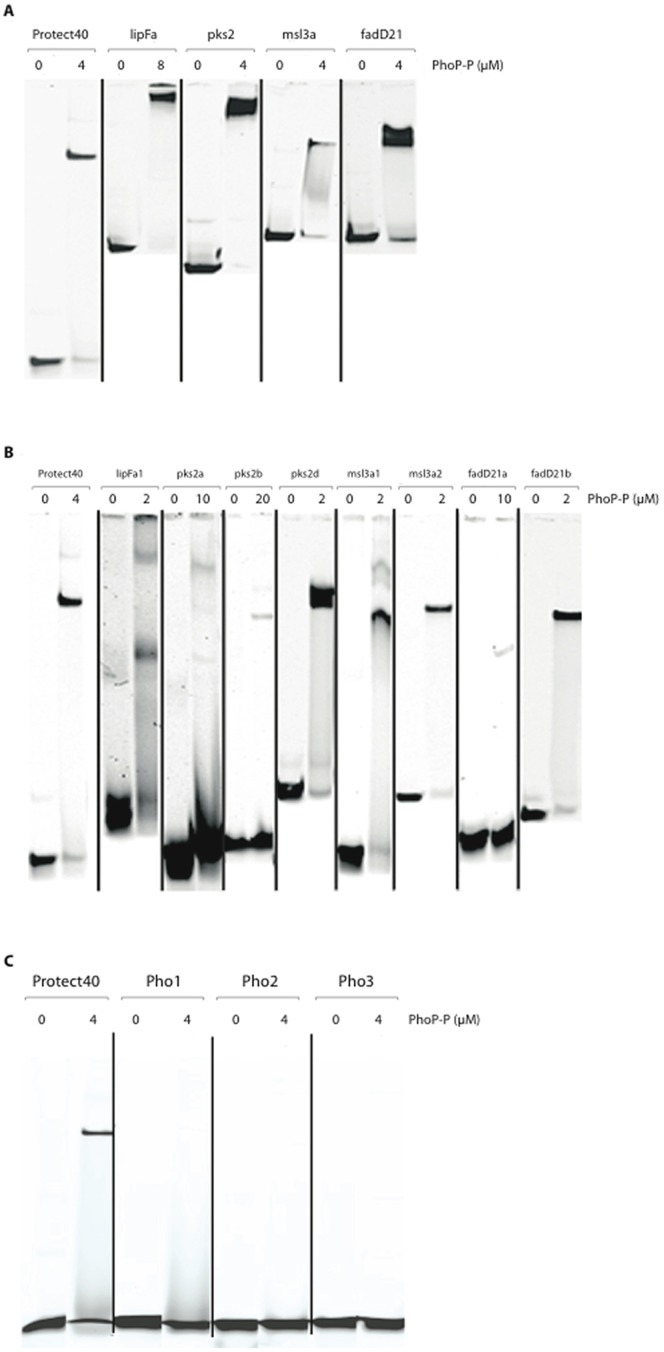
Electrophoretic mobility assays with the lipF, pks2, msl3 and fadD21 promoter fragments and PhoP-P. 2A. The large DNA fragments initially selected — lipFa (222 bp), pks2 (148 bp), msl3a (235 bp), and fadD21 (210 bp) — were incubated with PhoP-P in the presence of poly dI-dC at 10 µg/ml and run on a native polyacrylamide gel (8%), in 0.5× TBE buffer. Complete binding was assessed on the basis of the amount of shifted material for each fragment in the presence of 4 µM of PhoP-P (protein/DNA ratio: 100/1), except for lipFa, for which 8 µM PhoP-P was required (protein/DNA ratio: 200/1). 2B. Smaller DNA fragments — lipFa1 (68 bp), pks2a (59 bp), pks2b (40 bp), pks2d (104 bp), msl3a1 (45 bp), msl3a2 (83 pb), fadD21a (55 bp) and fadD21b (79 bp) — were designed and incubated with PhoP-P in the presence of poly dI-dC at 10 µg/ml. Protect40 (40 bp) was tested in all mobility shift assays, as a positive control [26]. 2C. Protect40 (40 bp) fragments with an altered sequence were used: Pho1 (DR1 altered sequence), Pho2 (DR2 altered sequence) and Pho3 (DR1 and DR2 altered sequences). Wild-type Protect40 (40 bp) was tested as a positive control [26].

We investigated whether PhoP bound directly to the DNA sequences upstream from *lipF*, *pks2*, *msl3* and *fadD21* identified in gene fusion studies, by carrying out electrophoretic mobility shift assays (EMSA) with phosphorylated PhoP protein (PhoP-P). In fact, previous studies have shown that PhoP must be phosphorylated for efficient binding to specific sites. We also carried out EMSA with the regulatory binding region of the PhoP gene itself, Protect40, as a positive control [29], [30]. Only positive bindings were shown in Fig. 2. We started by using PCR fragments of about 200 bp in size for EMSA (Table 1, Table 2, and Fig. 2A). For the *lipF* promoter region, we generated three 200 bp subfragments: lipFa (−596 to −375), lipFb (−394 to −168) and lipFc (−167 to +14). Only lipFa displayed a clear shift in electrophoretic mobility in the presence of PhoP-P (Fig. 2A and Fig.S1). For *msl3*, we generated two subfragments: msl3a (−282 to −49), which was about 200 bp in length, and msl3b (−67 to +28), which was about 100 bp long. Only msl3a displayed a clear shift in electrophoretic mobility in the presence of PhoP-P (Fig. 2A).

**Table 2:** List of DNA fragments used in this study.

Oligonucleotides	Sequence (5′-3′ position relative to the translation start)
**lipFa1** b	(−575) AGACGTACAGCAAACTCCCAGTCATACACCGATGCATTTCAAGGTGCCTGTGATCCCCTAAATGCAGG (−508)
**pks2a** b	(−52) GACACAGCTACATCGAAGGATTGCTAGTTAGGCTACGTATCGCCTCCGGCATTGGGCTT (+6)
**pks2b** b	(−95) GTGCCCCAGTAGCACAGCCGCTTAGAACTAAAGAGCCACC (−56)
**msl3a1** b	(−242) CGTCTGGTAGCGGCATGGCAACGGCCTGTGAGTTGGCTGGATAAT (−198)
**fadD21a** b	(−152) GGCGCCCCTGCGTACACCGACTTCGACTCTGCGTCAACCTGTTTCAGCACATGCA (−98)
**Protect40** b	(−70) GACTGTTAGCAGACTACTGGCAACGAGCTTTCAGGAATTA (−31)
**Pho1** b	(−70) GCTCAGGTAAAGACTACTGGCAACGAGCTTTCAGGAATTA (−31)
**Pho2** b	(−70) GACTGTTAGCAGACTTGAACGTCGGAGCTTTCAGGAATTA (−31)
**Pho3** b	(−70) GCTCAGGTAAAGACTTGAACGTCGGAGCTTTCAGGAATTA (−31)

b Fragments used for electrophoretic mobility shift assays.

The 147 bp (−86 to +60) pks2 and the 209 bp (−198 to +10) fadD21 fragments used in beta-galactosidase experiments also displayed a clear shift in electrophoretic mobility in the presence of PhoP-P (Fig. 2A). To address relative affinity differences between the DNA substrates, we varied the protein to DNA ratio to obtain a complete shift. The protein concentrations used ranged from 0 to 8 µM, for a constant DNA concentration of 40 nM. All five regulatory regions studied (*lipF*, *pks2*, *msl3*, *fadD21* and *phoP*) bound to PhoP-P resulted in the detection of higher molecular weight bands. For msl3a, fadD21 and pks2, a PhoP-P∶DNA ratio of 100∶1 was sufficient to obtain an almost complete shift, whereas a ratio of 200∶1 was required for lipFa. The various DNA segments thus have different affinities for PhoP-P (Fig. 2). A faint second band of higher molecular weight was observed for Protect-40, but not for the other targets analyzed here. All reactions were tested for the requirement for phosphorylation (Fig. 2). Non-phosphorylated assays are shown (Fig.S2). No significant binding was observed in absence of phosphorylation.

Using the data obtained with the large fragments, we carried out a sequence alignment analysis of all the fragments shown to bind PhoP-P. The regions similar to DR1/DR2 and DR3 previously identified in the *phoP* promoter [26] were observed.

**Figure 3:**
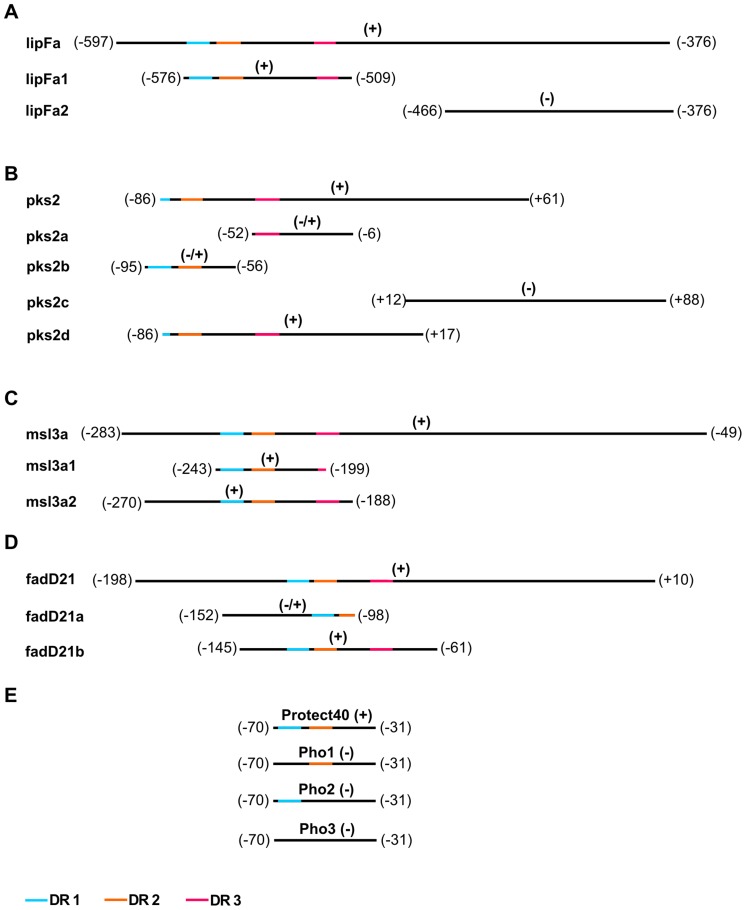
Schematic diagram of the DNA sequences tested in electrophoretic mobility assays. The DR1, DR2 and DR3 sites are indicated in blue, orange and red, respectively. Binding is indicated by (+), with (−) indicating an absence of binding and (−/+) indicating weak binding. Positions on the DNA regions are shown with respect to the translation start site, in brackets.

We applied trimming and walking techniques to the initial oligonucleotides to generate subsequent fragments of 50 to 100 bp in size (Fig. 2B, Fig. 3) and proceeded with PhoP-P binding.

The results from the EMSAs on the subfragments were analyzed in the context of the presence or absence of the DR repeats. In addition to lipFa, we also tested two shorter DNA regions for binding: lipFa1 (−575 to −508) and lipFa2 (−465 to −377). Binding was observed only with lipFa1, which contains DR1–3, at a PhoP-P concentration of 2 µM, but not for lipFa2. For *pks2*, we tested four additional shorter DNA regions: pks2a (−52 to +6), pks2b (−95 to −56), pks2c (+10 to +86) and pks2d (−86 to +15). Binding was observed for pks2d, which contains a partial DR1 and DR2–3 (at a PhoP-P concentration of 2 µM). Only very weak binding was observed for pks2a, which contained a DR3 sequence alone (at a PhoP-P concentration of 10 µM), and for pks2b, which contained only DR1 and DR2 sequences (at a PhoP-P concentration of 20 µM). pks2c which does not contain any of the DRs did not give any binding (Fig. 2B). We studied *msl3a* binding in more detail with two additional DNA fragments: msl3a1 (−242 to −198) and msl3a2 (−269 to −188). Binding was observed for both of these fragments; msl3a2 which contains the entire DR1–3 (binding observed at a PhoP-P concentration of 2 µM), whereas msl3a1 contains only the first 3 bp of DR3 (binding at a PhoP-P concentration of 2 µM). Finally, *fadD21* was studied by testing two additional DNA regions: fadD21a (−152 to −98), and fadD21b (−145 to −61). Binding was observed for fadD21b, which contained all three DR sequences (PhoP-P concentration of 2 µM). Only very weak binding was observed for fadD21a, which contained only DR1 and partial DR2 (PhoP-P concentration of 10 µM), as for pks2b, which also contained only DR1–2 (PhoP-P concentration of 20 µM). However, in assays of PhoP-P binding to Protect40, full binding was observed when we used a DR1–2-containing fragment (PhoP-P concentration of 4 µM). Interestingly, reducing the size of the DNA targets (45–102 bp) resulted in a decrease in the protein/DNA ratio (only 1∶50) required to obtain a shift, (Fig. 2B), showing that we indeed were able to increase the affinity as we narrowed down the targets to match the DRs.

The transcription initiation sites were determined for *lipF*, *pks2* and *msl3* in previous studies [31], [32]. This allowed us to locate DR1, DR2 and DR3 with regard to the transcription start.

We investigated the importance of the repeat motifs in sequence-specific DNA binding by PhoP, using oligonucleotides with altered nucleotide sequences Pho1, Pho2, Pho3 (Table 2, Fig. 2C and 3E) in place of DR1, DR2 or combined DR1/DR2 sequences, respectively whereby we removed one or all of the DR1–2 repeats. The Protect-40 wild-type sequence was used as a control (Fig. 2C). The Pho1–Pho3 altered sequences severely impaired PhoP-P binding. These results confirm that the binding of PhoP to the *phoP* promoter region is dependent on the direct repeat motifs.

### Characterization of the PhoP binding site by DNase I footprinting

**Figure 4:**
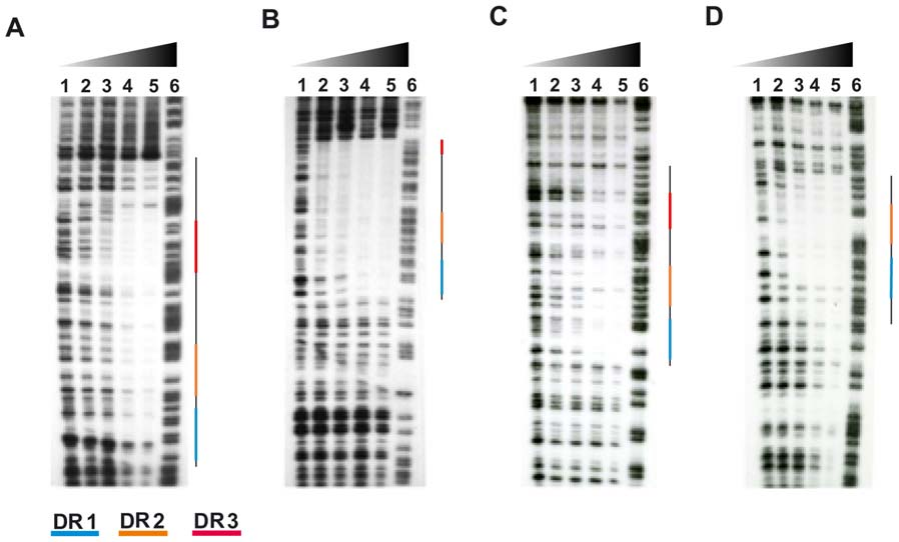
DNase I footprinting assay of the *pks2* and *msl3* promoter regions with PhoP-P. Radiolabeled PCR fragments corresponding to (A) pks2 (−136 to +62), (B) msl3 (−312 to −79), (C) fadD21(−194 to +7) and (D) lipF (−639 to −450) were used as DNA targets. Various amounts of PhoP-P were used with pks2 and msl3 (lane 1: 0; lane 2: 1.8 pmol; lane 3: 3.6 pmol; lane 4: 7.2 pmol and lane 5: 14.4 pmol) and were incubated with 0.2 pmol of DNA before DNase 1 digestion. For fadD21 and lipF different amount of PhoP-P were used ((lane 1: 0; lane 2: 1.8 pmol; lane 3: 18 pmol; lane 4: 180 pmol and lane 5: 900 pmol) and were incubated with 1 pmol of DNA before DNase I digestion. Lane 6: A+G Maxam and Gilbert reaction. Protected regions are indicated by a line with colored regions. The blue, orange and red segments correspond to the DR1, DR2 and DR3 sites, respectively.

We defined the DNA regions binding PhoP more precisely, by carrying out DNase I protection assays on the PhoP-P/DNA target complex. We used the *lipF*, *pks2*, *fadD21* and *msl3* promoter regions for these protection experiments. PhoP-P binding enhanced the protection of these four promoters against DNase I digestion (Fig. 4). The protected regions for *pks2, msl3 and fadD21* were approximately 50 to 70 bp long (Fig. 4). For *pks2* and *fadD21*, these regions include coverage of the DRs, the spacer region between them, with a complete DR3 motif. In the case of *msl3*, the DR3 is partially protected until position A7 known for being present in all the DRs (Table 3, [26]). The protected region of *lipF* is only 30–35 bp in length and the DR3 region is not protected by PhoP protein.

**Table 3:** Identification of the DR1–3 sites on the DNA promoters recognized byPhoP.

	DR1	DR2	DR3
*msl3*	TCTGGTAGC 8/9: 89%	CATGGCAAC 7/9: 78%	AATGTGTTC 5/9: 56%
*fadD21*	TGTTTCAGC 7/9: 78%	ATGCACAGC 6/9: 67%	ATGATCAGC 7/9: 78%
*lipF*	ACGTACAGC 8/9: 89%	ACTCCCAGT 6/9: 67%	CCTGTGATC 6/9: 67%
*pks2*	CCCAGTAGC 6/9: 67%	CCGCTTAGA 6/9: 67%	AGACACAGC 5/9: 56%
*phoP*	ACTGTTAGC 9/9: 100%	ACTGGCAAC 9/9: 100%	-
consensus sequence of *phoP*	A C T/G T/G T/G Py A Pu C		

For each DNA sequence shown to bind PhoP-P, sequences with a complete or partial match to the DR1, DR2 and DR3 sites were identified (underlined). The degree of conservation of the consensus sequence was calculated and is shown as the ratio of matching nucleotides to the consensus: % identity.

### DR sequence analyses

**Figure 5:**
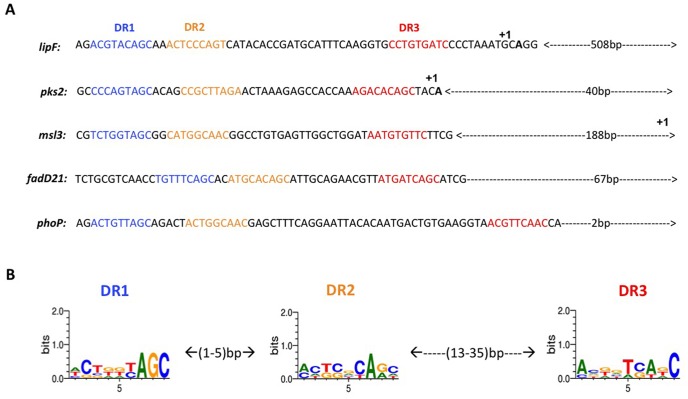
Identification of the DR1, DR2 and DR3 repeats in the upstream regions of the *phoP*, *lipF*, *pks2*, *msl3* and *fadD21* genes. (A) Nucleotide sequences and mapping of DR1, DR2 and DR3 sites. (B) Illustration of the consensus sequences of DR1, DR2 and DR3 obtained with MEME and Weblogo. The +1 indicate the transcription start site according to Richter *et al.*, (2007) for *lipF* and Goyal *et al.*, (2011) for *pks2* and *msl3*.

**Figure 6:**
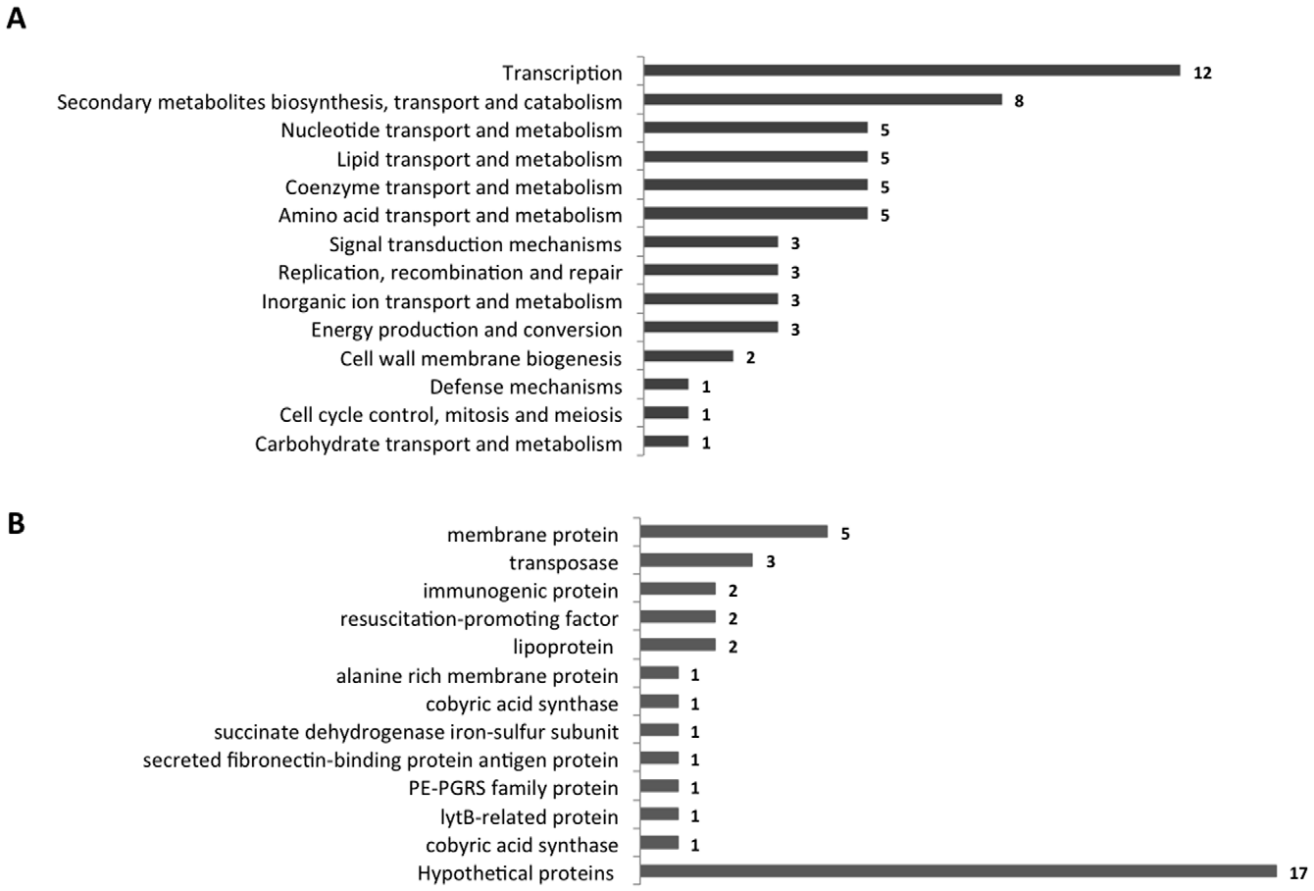
Distribution of the identified consensus sequences of DR1, DR2 and DR3 in the upstream regions of all *Mycobacterium tuberculosis* H37Rv genes. (A) Distribution according to the Clusters of Orthologous Groups (COG) classification. (B) Genes not listed in the COGs classification were classified on the basis of the functions listed in Tuberculist.

Using two different approaches (see Materials and Methods section), we identified three motifs matching the original DR1, DR2 and DR3 repeats of the PhoP promoter. These repeats are also present in all target regions upstream the *lipF*, *pks2*, *msl3* and *fadD21* genes (Fig. 5). The degree of sequence identity to the consensus of the canonical DR1–3 motifs of the *phoP* promoter region [23] ranged from 56% to 89% (Table 3). DR1 was the most conserved motif, followed by DR2, and then DR3. The DR1 and DR2 motifs were separated by 1 to 5 bp. By contrast, DR2 and DR3 were separated by 13 to 35 bp (Fig. 5). Taking this into account, we analyzed the distribution of the consensus sequences for each motif throughout the MTB genome. Motif configurations with only DR1 and DR2, or with DR1, DR2 and DR3 were observed in the upstream regions of 87 genes, accounting for 83.9% and 16.1% of total cases, respectively. The identified genes were analyzed according to the classes of the Clusters of Orthologous Groups (COGs) classification [29], [30] (Fig. 6A, Table S1). We further investigated, using the Tuberculist data, the function of genes that are not listed in COGs (38 genes, Fig. 6B). Some of the identified genes have already been reported to be regulated by PhoP on the basis of transcriptomic data [20], [21]. Based on the COG classification, we found that PhoP play a key role as a putative transcription regulator mostly for genes involved in transcription (21%) and Secondary metabolites biosynthesis, transport and catabolism (14%), lipid transport and metabolism (9%), and coenzyme transport and metabolism (9%) (Fig. 6A).

## Discussion

All living organisms have developed regulatory networks allowing them to survive in different environments, through rapid adaptation to hostile conditions and the conservation of energy by blocking pointless biochemical biosynthesis. TCS regulators, which have an environmentally responsive component, play a major role in this process. One of the advantages of this mode of control is that it allows the expression of complex traits or actions, such as membrane biosynthesis, host defences escape or adaptation, to be coordinated. This regulation results in changes to the patterns of expression of multiple genes [33], [34].

The MTB PhoP/PhoR TCS regulates the expression of more than 100 genes, based on differential gene expression data [20]. This includes genes encoding proteins involved in polyketide synthesis, for which differences in expression have been observed between PhoP null mutants and wild-type strains.

We aimed to identify genes directly regulated by PhoP. Transcriptome analyses have identified groups of genes regulated by PhoP, including genes encoding regulators that might function as intermediates in regulatory cascades [20], [21]. An analysis and comparison of these groups showed that they had only a few genes in common. This result reflects the problems involved in dealing with genes displaying low-level regulation and the importance of taking into account different experimental conditions and different strains genotypes. Indeed, as PhoP plays a key role in the regulation of several virulence factors, its activation varies with stressful conditions and the nature of the stimulus of the cognate sensor. We used a combination of genetic, biochemical and bio-informatics approaches to define PhoP binding sites as hallmarks of PhoP-regulated genes. We studied *lipF, msl3, pks2* and *fadD21*, which were shown to be upregulated by PhoP in genetic [29] and transcriptomic studies [20], [21]. However, there has been little evidence for the mechanism of regulation by PhoP in terms of DNA recognition sites, binding to selected genes or search for genes distributed throughout the genome with sub threshold regulation levels.

*M. smegmatis* has been used as a model strain to test *lipF, pks2, msl3*, and *fadD21 lacZ* gene fusions for the identification of promoters. The flanking regions of these genes were used for direct assessments of PhoP interaction by *in vitro* EMSA and DNase I protection assays. All experiments have been done using phosphorylated PhoP protein as unphosphorylated MTB PhoP binds weakly to oligonucleotides derived from the *lipF, pks2*, *msl3*, and *fadD21*, promoter sequences as previously described [31], [32], [35]. These observations are similar to those for PhoB from *E. coli*, in which PhoR and PhoB constitute a two-component system controlling phosphate uptake [35].

The first study on PhoP protein interaction with its own promoter revealed the presence of three direct repeat motifs (DRs) [26]. The first two direct repeats have been shown later to be sufficient for this interaction [30], [32]. More recently, the analyses of the promoter regions of *pks2* and *msl3* genes, revealed the presence of two DRs motifs that are needed for recognition by PhoP protein [31]. Nevertheless the motifs published by Goyal *et al.* (2011) are different from those that we identified. While the previously identified DRs regions are different from those reported in our study, the two works identified, overall, the same protected region. The main difference resides in the fact that we took in consideration the previously confirmed DRs regions identified in the promoter region of the *phoP* gene. This led us to clearly identify the presence of DR1–3 in some of the promoters that bound PhoP-P, and also allowed us to witness in some cases loss of binding when DR3 was missing. This is confirmed by our footprinting assays and gel shift experiments.

In this study, we show that for all studied genes, we were able to identify in their promoter regions three DRs namely DR1, DR2 and DR3. In addition, the DR3 repeat is essential for the binding of PhoP to *pks2* and *fadD21* DR1–2 repeats. This result is consistent with a potential cooperative interaction between DR3 and DR1–2. By comparing all identified DRs motifs we could suggest that DR3 assisted the binding to all PhoP-P-regulated sites showing low levels of similarity to *phoP*'s DR1 and DR2 sequences (Table 3). Moreover, the ratio of PhoP to DNA required to achieve this binding is variable, thus resulting in additional fine-tuning of expression for different genes under the control of PhoP.

The *lipF* promoter region contains, at positions −574, −563, and −530, sequences similar to the previously described DR1, DR2 and DR3 of PhoP. Richter et al. (2007) reported that the position of the transcription start was 511 bp upstream from the translation initiation site. Our results indicate that the lipF −10 site identified by Richter et al. (2007) overlaps with the newly assigned DR3 box described here. Based on the distance between the *lipF* DR1/DR2 sites and the transcription start site, we suggest that a tandem head-to-tail PhoP dimer, close to the −35, binds to the DR1/DR2 repeats that occupy adjacent same-DNA-face strands. The same configuration is also observed for *pks2*. A similar situation has been described for *E. coli* PhoB, which activates transcription by interacting with the sigma 70 subunit of the RNA polymerase in promoters in which the −35 sigma recognition element is replaced by the Pho box.

PhoP dimerization would bring the two copies of the DNA-binding domains close to each other, facilitating binding to DR1 and DR2, which are separated by only two nucleotides in this case. The presence of DR1/DR2 PhoP binding sites that overlaps with the −35 transcription region of *lipF* and *pks2* might be indicative of a class II activation mechanism according to which the regulator PhoP binds the region 4 of the sigma factor [36]. This configuration might result in the recruitment of the RNA polymerase to the promoter and subsequent induction of transcription start. The presence of DR1/DR2 PhoP binding site upstream the −35 transcription region for *msl3* might involve another mechanism of transcription activation that remains to be investigated.

With the use of various bioinformatics analyses, we fine-tuned the three consensus sequences for the individual DR1, DR2 and DR3 sites (Fig. 6). Only DR1 and DR2 were highly conserved in all analysed regions. We used these confirmed consensus sequences by taking into account the length of the spacer sequences between them to investigate the distribution of these motifs throughout the *Mycobacterium tuberculosis* genome, in order to identify all genes that could be potentially regulated directly by the PhoP/PhoR TCS. We found these motifs in the upstream regions of 87 genes, including genes having already been reported to be regulated by PhoP [21]. The classification of all identified genes into COG categories revealed a predominance of genes encoding proteins involved in transcriptional regulation (Fig. 6). This result reflects and confirms the role of *phoP* as a key player in the virulence of tubercle bacilli by controlling the activity of several genes. Indeed, PhoP has been shown to regulate the differential transcription of up to 600 genes in *E. coli* [37]. Genes encoding proteins involved in the biosynthesis of polyketide-derived lipids were also well represented, providing additional support for our approach. These genes have already been reported to be under the control of the phoP/phoR regulon [20], [21]. Some of the other genes identified were classified as involved in lipid biosynthesis pathways. This finding is consistent with previous studies showing that synthesis of the lipid components of the cell surface is regulated by PhoB in *E. coli*.

Our analysis provides information that cannot be obtained easily by transcriptomic approaches, because the stimuli of the PhoP cognate sensor have yet to be identified and because transcriptional regulators are often only weakly expressed. The identification of genes directly regulated by PhoP paves the way for the characterization of genes involved in pathogenicity. The role of these genes should be further investigated by genetic approaches and may be used as a starting point for the design of new drugs and vaccines.

## Materials and Methods

### Bacterial strains, media and growth conditions

*Mycobacterium tuberculosis* H37Rv [6], *M. smegmatis* mc^2^155 [38] and their derivatives were cultured at 37°C in Middlebrook 7H9 medium supplemented with ADC (0.5% bovine serum albumin, 0.2% dextrose, 0.085% NaCl, 0.0003% beef catalase; Difco) and 0.05% Tween 80 or on solid Middlebrook 7H10 medium supplemented with OADC (0.05% oleic acid, 0.5% bovine serum albumin, 0.2% dextrose, 0.085% NaCl, 0.0003% beef catalase; Difco). *Escherichia coli* cultures for cloning were grown in Luria-Bertani (LB) broth or on LB agar plates at 37°C. When required, antibiotics were added to the medium at the following concentrations: kanamycin (20 µg/ml), ampicillin (100 µg/ml). We added isopropyl-β-D-thiogalactopyranoside (IPTG) at a concentration of 0.5 mM and X-Gal at a concentration of 50 µg/ml, when required.

### Plasmid construction

For construction of the p*lipF*, p*pks2*, *pmsl3* and p*fadD21* promoter-*lacZ* fusions, we amplified the corresponding fragments from *M. tuberculosis* H37Rv genomic DNA by PCR with the primers shown in Table 1. The PCR products were digested with *Bam*HI and *Kpn*I and inserted into the corresponding sites in the pJEM15 *E. coli*-mycobacterial shuttle plasmid [39]. We used pJEM31-PAN containing a promoter sequence, PAN, isolated from *M. paratuberculosis* as a positive control. This promoter lies adjacent to and does not overlap the 3′ end of an IS900 mobile genetic element [28]. This plasmid was used to transform *M. smegmatis* mc^2^155. The strains were grown at 37°C in 7H9 medium supplemented with kanamycin (20 µg/ml), until they reached an optical density at 600 nm (OD_600 nm_) of 0.6–0.8. The cells were collected by centrifugation and β-galactosidase activity in the cell extract was evaluated to determine the levels of transcription from the various promoter regions studied (*lipF*, *pks2*, *msl3*, and *fadD21*).

### Quantification of beta-galactosidase activity

Mycobacterial strains were grown to the exponential growth phase (OD_600 nm_ = 0.8). The cultures were centrifuged, and the pellets were washed with phosphate-buffered saline. The cells were resuspended in 1 ml Z buffer (0.06 M Na_2_HPO_4_, 0.04 M NaH_2_PO_4_, 0.01 M KCl, 1 mM MgSO_4_ and 0.05 M β-mercaptoethanol pH 7 at 25°C and sonicated to generate a cell extract. The chromogenic substrate o-nitrophenyl-D-galactoside (Sigma, France) was added to the cell extracts at a final concentration of 0.66 mg/ml. The mixtures were incubated at 28°C for 1 hour and the enzymatic reactions were stopped by adding 0.29 M Na_2_CO_3_. The A_420 nm_ of the supernatant was determined, and β-galactosidase activity was calculated in Miller units, according to the following formula: β-galactosidase activity = (1000×A_420 nm_)/(time (min)×aliquot volume (ml)×OD_600 nm_).

### Electrotransformation of mycobacteria

50 ml culture of bacteria was grown to an OD_600 nm_ of 0.6 to 0.8. The cells were collected by centrifugation, washed twice in 10% glycerol and resuspended in 2 ml of 10% glycerol. Aliquots (400 µl) were electroporated with vector DNA in 0.2 cm-path length cuvettes (Bio-Rad), with a single pulse (2.5 kV, 25 µF, 1000 Ω). Cells were transferred to 1 ml of 7H9-ADC-0.05% Tween 80 and incubated for 2 hours at 37°C.

### Production, purification and phosphorylation of PhoP

The PhoP protein was produced in *E. coli* Bl21(DE3) pLysS (Agilent Technologies), from the pET15b expression plasmid (Novagen, Merck Chemicals France) [30]. PhoP overproduction was induced by adding 1 mM IPTG (Euromedex, France) to LB medium and incubating for 3 hours at 30°C. The cells were collected by centrifugation at 5,500 rpm (SLA-4000 rotor, RC5c (Sorvall) centrifuge) for 30 min and resuspended in 50 mM Tris-HCl pH 8.0, 1 M NaCl, 5 mM KCl, 10% glycerol, 5 mM imidazole, 100 µM phenyl methyl sulfonyl fluoride (PMSF) (Buffer A). The cells were lysed by passage through an Emulsiflex-C5 (Avestin Europe) at 4°C. PhoP was purified on a 5-ml HiTrap Chelating HP (GE Healthcare) affinity column loaded with 0.5 M Ni_2_SO_4_ and equilibrated with Buffer A, on an AKTA-prime FPLC machine (GE Healthcare). The histidine tag was cleaved overnight at 4°C, with a Thrombin CleanCleavage kit (Sigma Aldrich). The released PhoP was further purified with a second passage through the 5-ml HiTrap Chelating HP Ni-affinity column. Pure protein (>95%, based on SDS-PAGE) was obtained after several rounds of FPLC, and stored at −80°C in 50 mM Tris-HCl pH 8, 150 mM NaCl, 5 mM KCl, and 20% glycerol.

Aliquots of 200 nmoles of PhoP were incubated for 90 minutes at 30°C with 50 mM acetyl phosphate (AcP) (Sigma Aldrich) in 100 mM Tris-HCl pH 7.0, 10 mM MgCl_2_, 150 mM KCl, to obtain phosphorylated PhoP (PhoP-P).

### DNA fragment synthesis and purification

Double-stranded DNA fragments (ds-DNAs) were labeled at the 5′ end by PCR with commercially synthesized 5′-labeled IRD700 or DY682 (Eurofins MWG Operon, Germany) primer and the various templates described in Table 1, 2.

ds-DNAs were purified by electrophoresis in 8% polyacrylamide gels, electroeluted in 0.5× TBE (Biosolve, Netherlands), through 10 kDa MWCO (molecular weight cut off) dialysis tubing (Spectra/Por, Spectrum Labs, USA), and dried on a Speed Vac. The DNAs were resuspended in 60 mM sodium acetate pH 5.4, precipitated in 100% ethanol, washed in 70% ethanol and recovered in 10 mM Tris pH 7.8, 0.1 mM EDTA (Gibco). The DNA concentrations were deduced from A_260 nm_ before use or storage.

### Electrophoretic mobility shift assay (EMSA)

Aliquots of 40 nM labeled ds-DNAs were separately mixed with various concentrations of PhoP or PhoP-P, in a final volume of 30 µl in the presence of poly dI-dC at 10 µg/ml. The mixture was dialyzed in several steps against 50 mM Tris-HCl, pH 8.0, 1 mM dithiothreitol (DTT), 1 mM EDTA, 100 µM PMSF, with decreasing concentrations of NaCl (750 mM, 250 mM, 150 mM respectively), at 4°C. The DNA/PhoP(-P) complex obtained at the end of dialysis was mixed with 3 µl of gel loading buffer (0.1% bromophenol blue and 40% sucrose), loaded onto a 20-cm×20-cm 8% native polyacrylamide gel and subjected to electrophoresis at 180 V/cm for 3 hours at 18°C, with 0.5× TBE used as the running buffer. The gels were then transferred onto blotting paper (Whatman 3MM CHR) and scanned on an Odyssey® Infrared Imaging System at 700 nm (Li-cor Inc, NE, USA).

### DNaseI footprinting experiments

DNA probes were prepared by amplifying fragments of the *lipF*, *pks2*, *msl3* and *fadD21* upstream regions by PCR with the lipFDF/lipFDR, msl3DF/msl3DR pks2DF/pks2DR and fadD21DF/fadD21DR primers, respectively, using *M. tuberculosis* chromosomal DNA as the template. In each case, the 5′ end of the forward primer (lipFDF, msl3DF, pks2DF and fadD21DF) was labeled with [γ-^32^P]ATP, with T4 polynucleotide kinase. Before the DNA binding reaction, PhoP-P was obtained as described above and dialyzed against a buffer containing 20 mM Tris pH 8, 1 mM EDTA, 200 mM NaCl, 50% glycerol. DNase I footprinting reactions were performed as previously described [25]. Briefly, 0 to 15 pmol of PhoP-P was mixed with 0.2 pmol of DNA and incubated with DNase 1 at room temperature (∼24°C) for 1 min. The samples were analyzed by electrophoresis on a 6% polyacrylamide gel containing 7 M urea. Maxam and Gilbert sequencing ladders (G+A) were also loaded on the same gel.

### Sequence analysis

We narrowed down the DNA sequences potentially recognized by PhoP-P (to ∼60 bp) by EMSA, with iterative sequence alignment to DR1 and DR2 (EMBOSS package, ClustalW). The results were confirmed by footprinting. The resulting sequence datasets were then analyzed further, by two different approaches. In the first, we used a Python script (this study) to search the identified regions for motifs identical and similar (no more than three nucleotides of difference) to those already identified in the upstream region of the PhoP gene [20], [26], [32]. We then analyzed these regions with the MEME (Multiple Em for Motif Elicitation) program [40], which uses the expectation–maximization (EM) algorithm for iterative improvement of a model of the motif. Finally, we constructed consensus motif sequences with WebLogo 3 [41], [42]. The distribution of these consensus sequences in the *Mycobacterium tuberculosis* genome was analyzed with a Python script (this study) by scanning the 800 bp regions immediately upstream all *M. tuberculosis* H37Rv genes.

## Supporting Information

Figure S1 **Electrophoretic mobility assays with the lipFb, lipFc, msl3b, lipFa2 and pks2c fragments and PhoP-P.** lipFb (227 bp), lipFc (182 bp), msl3b (98 bp), lipFa2 (89 bp) and pks2c (77 bp) fragments were incubated with PhoP-P in the presence of poly dI-dC at 10 µg/ml and run on a native polyacrylamide gel (8%), in 0.5× TBE buffer. Each fragment was incubated in the presence of 8 µM PhoP-P, with the exception of msl3b, for which 10 µM PhoP-P.
(EPS)

Figure S2 **Electrophoretic mobility assays for the lipFa, pks2, msl3 and fadD21 fragments with phosphorylated (P) and unphosphorylated (NP) PhoP.** The large DNA fragments initially selected — lipFa (222 bp), pks2 (148 bp), msl3a (235 bp), and fadD21 (210 bp) — were incubated with PhoP-P and unphosphorylated PhoP in the presence of poly dI-dC at 10 µg/ml and run on a native polyacrylamide gel (8%), in 0.5× TBE buffer. Each fragment was incubated in the presence of 4 µM PhoP-P and PhoP, with the exception of lipFa, for which 8 µM PhoP-P and PhoP was required.
(TIFF)

Table S1 **List of genes with DR1, DR2 and DR3 in their upstream regions according to** **Figure 5** **.** First Excel spreadsheet: DR1+DR2; Second spreadsheet: DR1+DR2+DR3.
(XLS)
