# Supplementary figures and images for: Identification of DNA Binding Motifs of the Mycobacterium tuberculosis PhoP/PhoR Two-Component Signal Transduction System
Source: PLoS One. 2012 Aug 7;7(8):e42876. doi: 10.1371/journal.pone.0042876 (PMC3413638; doi:10.1371/journal.pone.0042876)

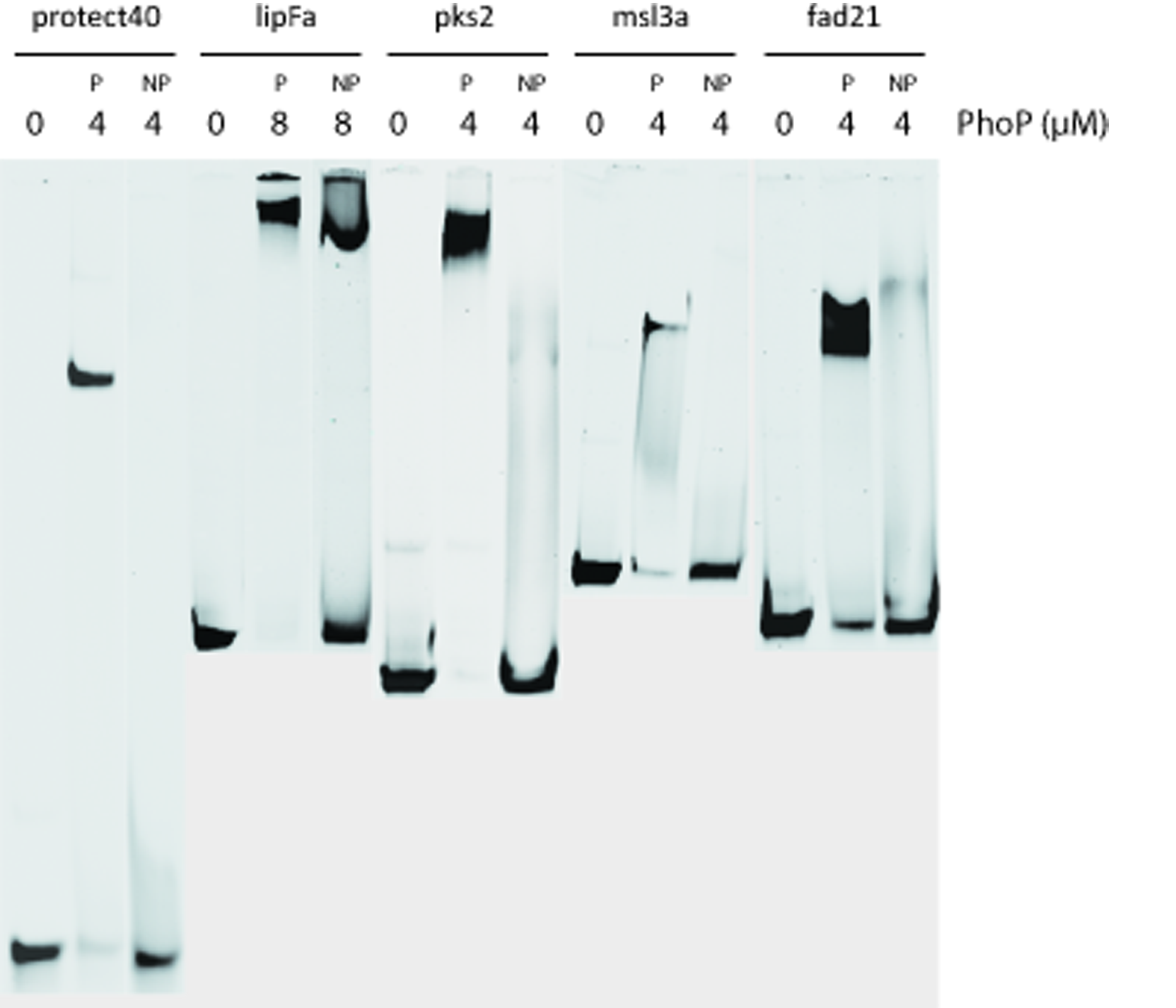

Supplement: Figure S2 — Electrophoretic mobility assays for the lipFa, pks2, msl3 and fadD21 fragments with phosphorylated (P) and unphosphorylated (NP) PhoP. The large DNA fragments initially selected — lipFa (222 bp), pks2 (148 bp), msl3a (235 bp), and fadD21 (210 bp) — were incubated with PhoP-P and unphosphorylated PhoP in the presence of poly dI-dC at 10 µg/ml and run on a native polyacrylamide gel (8%), in 0.5× TBE buffer. Each fragment was incubated in the presence of 4 µM PhoP-P and PhoP, with the exception of lipFa, for which 8 µM PhoP-P and PhoP was required. (TIFF) [file pone.0042876.s002.tiff]
